# A Novel Prodrug Approach for Central Nervous System-Selective Estrogen Therapy

**DOI:** 10.3390/molecules24224197

**Published:** 2019-11-19

**Authors:** Katalin Prokai-Tatrai, Laszlo Prokai

**Affiliations:** Department of Pharmacology and Neuroscience, University of North Texas Health Science Center, Fort Worth, TX 76107, USA; laszlo.prokai@unthsc.edu

**Keywords:** antioxidant, estrogens, bioprecursor prodrug, brain, cognition, CNS-selective estrogen therapy, cognition, depression, DHED, glaucoma, menopause, neuroprotection, *para*-quinol, photoreceptor, retina, stroke

## Abstract

Beneficial effects of estrogens in the central nervous system (CNS) results from the synergistic combination of their well-orchestrated genomic and non-genomic actions, making them potential broad-spectrum neurotherapeutic agents. However, owing to unwanted peripheral hormonal burdens by any currently known non-invasive drug administrations, the development of estrogens as safe pharmacotherapeutic modalities cannot be realized until they are confined specifically and selectively to the site of action. We have developed small-molecule bioprecursor prodrugs carrying the *para*-quinol scaffold on the steroidal A-ring that are preferentially metabolized in the CNS to the corresponding estrogens. Here, we give an overview of our discovery of these prodrugs. Selected examples are shown to illustrate that, independently of the route of administrations and duration of treatments, these agents produce high concentration of estrogens only in the CNS without peripheral hormonal liability. 10β,17β-Dihydroxyestra-1,4-dien-3-one (DHED) has been the best-studied representative of this novel type of prodrugs for brain and retina health. Specific applications in preclinical animal models of centrally-regulated and estrogen-responsive human diseases, including neurodegeneration, menopausal symptoms, cognitive decline and depression, are discussed to demonstrate the translational potential of our prodrug approach for CNS-selective and gender-independent estrogen therapy with inherent therapeutic safety.

## 1. Introduction

**Figure 1:**
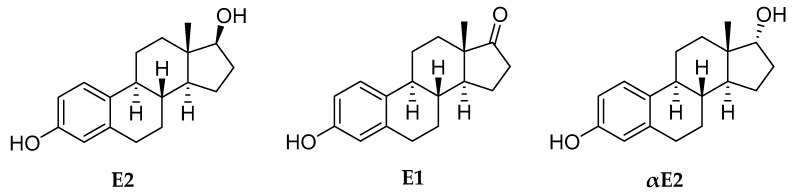
Chemical structure of 17β-estradiol (E2), estrone (E1) and 17α-estradiol (αE2).

Estrogens are increasingly recognized as neurosteroids of crucial importance, both in biological and medical contexts. Many neurological, psychiatric and neurodegenerative diseases that have been linked to deprivation of the human CNS, respond to or may be prevented by treatments with these hormones—most notably with the most potent human estrogen, 17β-estradiol (E2, Figure 1) [1,2,3]. Therefore, estrogen-based therapies have promised remedies for numerous CNS maladies in myriads of preclinical and clinical evaluations with therapeutic targets, including symptoms commonly associated with surgical and natural menopause [4,5], neurodegenerative diseases affecting the brain [6,7,8], trauma and stroke [9,10], psychiatric disorders [11], cognitive decline [12], as well as various ocular neurodegenerations [13,14]. On the other hand, current estrogen therapies cannot be used safely to treat most of these estrogen-responsive CNS conditions because of detrimental peripheral side-effects associated with the direct administration of estrogens. These include increased risk of breast cancer [15], thromboembolism, coronary heart disease and stroke [16,17], owing to significant estrogen levels in the circulation from the exogenously applied hormones. In fact, the controversial Women Health Initiative (WHI) study, which was a large placebo-controlled trial assessing the risks and benefits of menopausal hormone therapy (MHT), first published its findings associated with MHT on the increased risks of invasive breast cancer and cardiovascular liability that eventually led to its abrupt termination in 2002 [18]. MHT is based on estrogens to treat climacteric symptoms such as hot flushes and depression. A synthetic progesterone derivative (i.e., a progestin) is also necessary for women with intact uterus to provide endometrial protection against estrogen-induced hyperplasia, which can lead to cancer [19].

MHT was originally termed “hormone replacement” therapy—even though E2 lost with aging or pathological processes was rarely used. Rather, the so-called conjugated equine estrogens (CEE) extracted from pregnant horse urine have been the mainstream estrogens in this regard. This extract comprises a variety of estrogens, but not E2. CEE is mainly a mixture of sodium sulfate salts of estrone (E1, Figure 1) and the non-human equilin with concomitant components, such as 17α-estradiol (αE2, the 17C-epimer of E2, Figure 1), among many other non-human estrogens. Similarly, a progestin such as medroxyprogesterone acetate has also been included in MHT. However, CEE and progestins have different absorption, distribution, metabolism, excretion and toxicity (ADMETox) profiles [20] than endogenous hormones, which may have also played a significant role in triggering the adverse side-effects of MHT recognized by WHI. It is noteworthy that progestins are also believed to be detrimental to breast [21] and brain health [22]; nevertheless, they are necessary to protect the endometrium against unopposed circulating estrogens [15]. While subsequent data re-analyses somewhat toned down the unexpected negative outcomes of WHI [19], much controversy remains surrounding the risks and benefits of currently available MHT. Yet, estrogens are still the most effective treatment options for counteracting debilitating menopausal symptoms that can lead to a significantly diminished quality of life [23].

On the other hand, any currently approved estrogen or estrogen + progestin therapy for MHT produces significant circulating estrogen levels, leading to the unwanted peripheral hormonal liability described above, independently of the type of hormones, dosage form and duration of treatment. Feminization, including gynecomastia in men [24,25], is also a serious drawback of estrogen therapy for a certain population of prostate cancer patients who suffer from similar symptoms (hot flushes, cognitive decline, depression, etc.) as postmenopausal women. These symptoms also are due to estrogen deprivation of their brains, as testosterone is lost after surgical or chemical castration. In men and males of other species, brain estrogens are formed from androgens by local aromatase or synthesized de novo from cholesterol [24].

## 2. Estrogens as CNS Agents

Besides the already-mentioned thermoregulation, estrogens’ numerous beneficial actions in the CNS also include enhancing learning and memory and stabilizing mood, as well as protection against a wide variety of noxious stimuli leading to neurodegenerative processes [26,27,28]. The latter is especially important, as neuroprotection of the CNS is clearly a pressing unmet medical need. The pathophysiology of neurodegeneration is complex and multi-factorial in nature; therefore, development of pleiotropic, broad-spectrum neuroprotective agents impacting multiple mechanisms rather than a single druggable target is highly desired [26]. Estrogens, especially E2, possess such therapeutic properties and, therefore, are ideally suited as broad-spectrum neuroprotectants. The beneficial effects of estrogens on neuronal health come from a synergistic combination of genomic and non-genomic actions [26] that are not restricted to the brain but also important in ocular neuroprotection [13]. These benefits are many, and include prevention and reduction of inflammation, stabilization of mitochondrial membrane potential, elimination of free radicals and, thus, reduction of oxidative stress — all are critical contributors to collectively thwart both the initiation and progression of neuronal cell death [26]. However, the so-called “non-feminizing” estrogens are devoid of genomic actions because the phenolic OH is unavailable for binding to the nuclear estrogen receptors (ERs, present in ERα and ERβ isoforms) [29]. This important functional group is either blocked by a covalent derivatization or shielded by bulky neighboring substituent(s) [30]. As such, these estrogen derivatives cannot deliver the full benefits of estrogens as neurotherapeutic agents, and their extremely high lipophilicity also presents a formidable obstacle for practical drug development [29,30]. The importance of genomic actions of estrogens in the CNS is not surprising, considering that ERα and ERβ are abundantly expressed throughout the CNS, including the retina [26].

**Figure 2:**
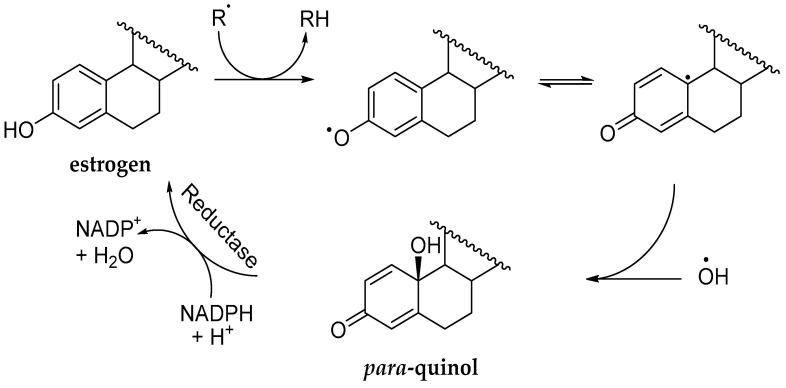
A novel antioxidant cycle for estrogens through the formation of *para*-quinol upon, e.g., chain-breaking H-atom transfer to a free radical (R^•^), leading to estrogen-derived phenoxyl radical that scavenges ^•^OH. The *para*-quinol is then reduced back to the parent hormone by an enzyme-catalyzed process involving the reduced form of nicotinamide adenine dinucleotide phosphate (NADPH) as a cofactor to rejuvenate the antioxidant estrogen.

The direct free radical scavenging ability of estrogens, a significant non-genomic action, is unique among neurosteroids, as only estrogens possess the phenolic A-ring that also renders them simple phenolic antioxidants [31,32,33]. This ability prominently contributes to the overall beneficial effects of estrogens in the CNS. Our investigation on how estrogens can provide a “chemical shield” against free radicals [34] led us to the development of the very first preclinical CNS-selective estrogen therapy approach discussed here [35,36,37]. As shown in Figure 2, in this endeavor we have recognized that, upon direct hydroxyl radical (^•^OH) capture, the phenolic A-ring of an estrogen undergoes oxidative dearomatization to *para*-quinol. The latter is then rapidly reduced back by an enzyme-catalyzed process to the corresponding estrogen [35]. This antioxidant cycle essentially regenerates the hormone, which can then exerts its well-orchestrated genomic and non-genomic actions for curing, curtailing, or treating estrogen-responsive and centrally regulated maladies [26,36,37].

This “Prokai cycle” [38] implies that an estrogen in the CNS does not have to be used up, because it can be rejuvenated after it has fulfilled its protective role against free radicals and, therefore, a very small concentration of estrogen at the site of action ought to be sufficient to set off this cycle. In simple terms, an estrogen is a “mop” that soaks up the most toxic free radicals before they can produce neurotoxicity. But when the mop is saturated (i.e., estrogen turned into *para*-quinol), the CNS quickly comes to the rescue to wring out the mop effectively and, thus, to turn the *para*-quinol back into estrogen and make it useful again.

In spite of impressive basic science and translational preclinical data, as well as epidemiological observations showing the potential strength of E2-based neurotherapeutic interventions, clinical applications can only be realized when therapeutic actions are restricted specifically and selectively to the CNS to assure therapeutic safety [13,23,26,35,36,37]. Development of CNS-selective estrogen therapies, however, has to measure up to daunting challenges, and only prodrug strategies have delivered promising progress to achieve this goal thus far.

## 3. Simple Prodrugs for CNS Delivery of Estrogens

Prodrugs are inert derivatives of therapeutic agents converted to the latter by enzymatic and/or chemical transformations in the body [39]. They represent one of the most versatile tools for medicinal chemists to resolve problems, associated, e.g., with a (parent) drug’s insufficient physicochemical properties, which often prevents efficacious drug delivery into the intended site of action, as most drugs reach the CNS through circulation by passive transport through the blood–brain barrier (BBB) [40].

The classical/simple prodrug approach considers the covalent attachment of property-modifying promoiety(ies) that can be removed in vivo once the prodrug has fulfilled its intended purpose. As mostly ester, carbamate or amide-types of prodrugs have been created due to synthetic simplicity, organ-targeting is difficult to achieve because of the ubiquitous distributions of enzymes responsible for prodrug metabolism to the parent agents. For example, simple prodrugs of E2 such as the clinically used E2-valerate, -acetate and -benzoate cannot achieve brain targeting, since bioactivation of these ester prodrugs occurs by non-specific hydrolysis via esterases abundantly expressed all over the body. When the water-soluble 17-*N*,*N*-dimethylaminobutyl ester prodrug of E2 was administered intranasally to bypass the formidable BBB, a very large concentration of E2 (ng/mL level) was present in the circulation, although a high level of E2 was also produced in the cerebrospinal fluid [41].

Targeted pharmacotherapy with prodrugs can only be achieved with site-directed delivery. This may be realized by, e.g., receptor-mediated enzyme prodrug systems, or site-specific facile bioactivation of the prodrug to the pharmacological active parent agent by enzymes preferentially expressed in the CNS [36,37,42,43]. An alternative strategy is to use promoieties that can undergo either reduction [44] or oxidation [45] to permanently-charged species within the brain to restrict their efflux back into the circulatory system, while these ionic species have been proposed to quickly excrete from the periphery by the renal route. The best-known concept that relies on an oxidative lock-in mechanism for enhanced brain delivery is the prodrug concept termed chemical delivery system (CDS) that utilizes dihydropyridine-based promoieties [45,46]. These can easily be oxidized to the corresponding pyridiniums on the analogy of NAD(P)H ↔ NAD(P)^+^. Mostly ester- and carbamate-type of CDSs have been prepared; therefore, hydrolases remove the oxidized promoiety (pyridinium) in the brain to release the parent drug at the intended site of action. This conceptually interesting approach suffers, however, from serious limitations, including but not limited to chemical tractability problems due to the well-known extreme sensitivity of dihydropyridines to oxidation, which is actually utilized for entrapping the CDS into the organ yet makes chemical synthesis, purification and formulation cumbersome [46].

The best-known application of this concept has been for the delivery of E2 into the brain. The E2-CDS provides a sustained E2 release within the brain from the oxidized E2-CDS, but its administration also produces at the same time a profound and sustained E2 level in the blood [46,47]. For example, even after a single dose of E2-CDS, several days are needed until the E2-CDS-derived E2 is eliminated from the circulation. Consequently, a significant increase in wet uterine weight relative to the control group could also be measured owing to the large increase in circulating E2 [46]. The large increase in wet uterine weight due to exogenously applied estrogens, the so-called uterotrophic effect, is a frequently used sensitive assay for the initial survey of peripheral estrogenicity [36]. For the first time, we have also shown that the increased wet uterine weight of the E2-CDS-treated animals was clearly associated with a large (1–3 ng/g) E2 content [46]. Considering the detrimental effect of unopposed E2 on the endometrium [19], because of the significant blood E2 level even upon a single dose of E2-CDS, this prodrug approach cannot be asserted, therefore, as a brain-selective or targeted brain-delivery of the hormone causing no peripheral burden.

## 4. Bioprecursor Prodrugs for CNS-Selective Estrogen Therapy

**Figure 3:**
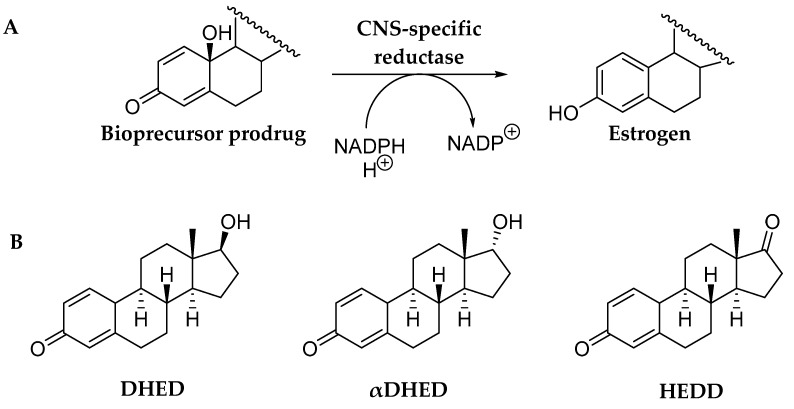
(**A**) Schematic illustration of CNS-specific reductive bioactivation of bioprecursor prodrugs shown in panel **B** by an NADPH-dependent short-chain dehydrogenase to the corresponding estrogen (E2, αE2 or E1). (**B**) Chemical structures of bioprecursor prodrugs of estrogens: 10β,17β-dihydroxyestra-1,4-dien-3-one (DHED) for E2; 10β,17α-dihydroxyestra-1,4-dien-3-one (αDHED) for αE2, and 10β-hydroxyestra-1,4-dien-3,17-dione (HEDD) for E1.

Unlike the classical prodrugs, bioprecursor prodrugs do not have auxiliary promoiety(ies) but the drug molecule itself is modified bioreversibly [36]. This type of prodrug undergoes Phase I metabolism (such as oxidation, reduction, decarboxylation, etc.) to regenerate the parent agent from the inert prodrug. One of the great advantages of bioprecursor prodrugs over classical prodrugs is that promoiety-related toxicity or innate pharmacological effects are eliminated. As mentioned previously and shown in Figure 2, our investigation on the chemical shield erected by estrogens upon capturing hydroxyl radicals led us to the recognition of estrogen-derived *para*-quinols that are enzymatically reduced back to the corresponding estrogens. This facile conversion, however, occurs only in the CNS (Figure 3A) rendering these *para-*quinols CNS-selective bioprecursor prodrugs of their parent estrogens. The chemical structures of 10β,17β-dihydroxyestra-1,4-dien-3-one (DHED), 10β,17α-dihydroxyestra-1,4-dien-3-one (αDHED), and 10β-hydroxyestra-1,4-dien-3,17-dione, (HEDD) are shown in Figure 3B as prodrugs for E2, αE2 and E1, respectively [35,36,37]. Estrogen-derived *para*-quinols can easily be synthesized through a one-step oxidation of the phenolic A-ring of the estrogens [48,49].

**Figure 4:**
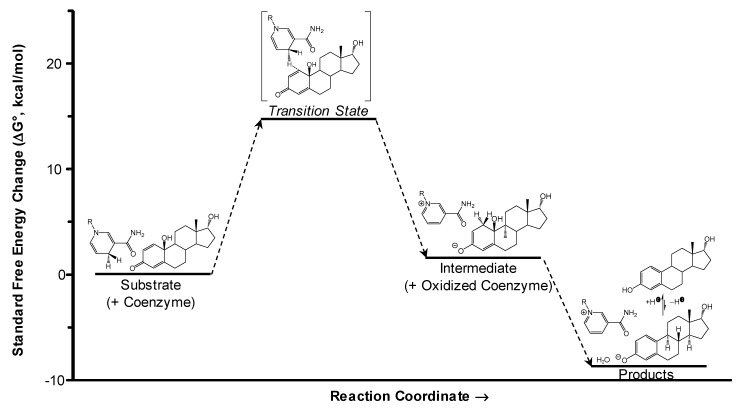
A mechanistic model for αDHED’s bioactivation to αE2. Semi-empirical quantum-chemical calculations (PM7 parametrization) were applied to the complete prodrug structures and a mimic of NAD(P)H ⇌ NAD(P)^+^ (R = CH_3_), and simulating solvation with water through the conductor-like screening model (COSMO). Reproduced with permission from Reference [37]; copyright American Chemical Society.

A computer-aided mechanistic study on these bioprecursor prodrugs’ reductive bioactivations to the corresponding estrogens in the CNS (Figure 3A) is shown through the example of αDHED in Figure 4 [37]. This process involves a transition state in which hydride transfer from NADPH to the C3 of the A-ring occurs, followed by water elimination and subsequent re-aromatization to αE2. The overall process is exergonic (hence spontaneous) based on the calculated standard Gibb’s free energy change (ΔG^0^). Thus far, DHED (Figure 3B) has been studied most extensively in preclinical animal models of E2-responsive maladies [23,36,50,51]. The unprecedented CNS selectivity of our approach in terms of prodrug bioactivation has also recently been confirmed independently from our laboratory [52,53]. We have also unambiguously shown the CNS-selective bioactivations of HEDD and αDHD to E1 and αE2, respectively [35,37].

**Figure 5:**
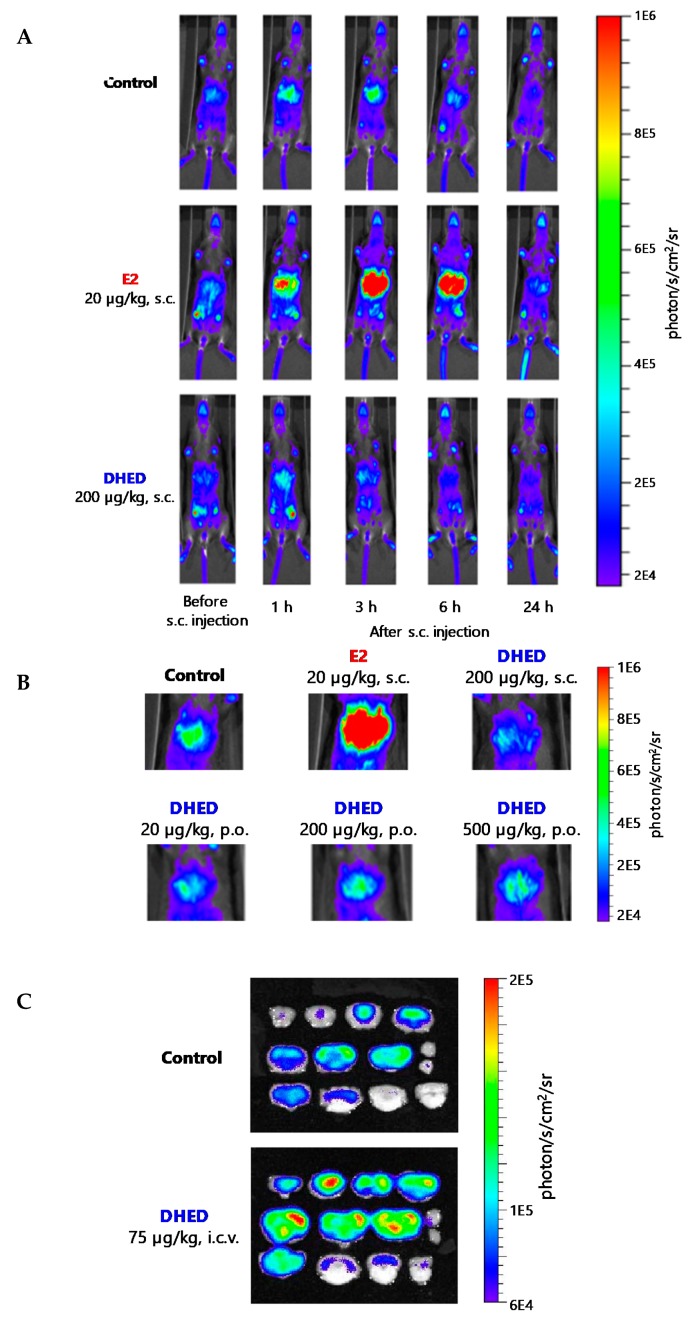
Bioluminescence imaging in repTOP ERE-Luc mice. (**A**) Time course analysis of in vivo bioluminescence after subcutaneous (s.c.) administrations of E2 (20 μg/kg) and DHED (200 μg/kg). (**B**) In vivo bioluminescense images from the hepatic area 3 h after treatment with vehicle (negative control, p.o.), E2 (20 μg/kg, s.c., positive control) and DHED (200 μg/kg s.c., as well as 20 μg/kg, 200 μg/kg and 500 μg/kg p.o.). (**C**) Bioluminescense in brain slices after intracerebroventricular (i.c.v.) injection of vehicle and DHED (75 µg/kg). Reproduced with permission from Reference [36]; copyright American Association for the Advancement of Science.

One of the proof-of-concept methodologies for lack of peripheral exposure to E2 upon DHED administration was done by bioluminescence imaging in repTOP ERE-Luc mice, in which luciferase expression depends on ER’s transcription [54]. Figure 5A shows that even a 10-fold higher dose of DHED treatment compared to that of E2 did not activate the estrogen-responsive element (ERE) luciferin (Luc) reporter gene construct and, thus, did not generate bioluminescence in the periphery, whereas a significantly lower dose of E2 produced a profound increase in bioluminescence with the maximum effect obtained about 3 h after drug treatment [36]. At this selected time point, ER activation could not be seen in the estrogen-sensitive hepatic area (Figure 5B), even with increasing doses of oral DHED, due to lack of peripheral metabolism to the parent E2. At the same time, E2 produced a significant ER activation, even at a significantly lower dose. However, DHED produced a profound ER activation, owing to its facile metabolism to E2 within the brain (Figure 5C). The selective formation of E2 and, thus, the lack of estrogen exposure after systemic DHED administration, were also shown by traditional and (through the use of the stable-isotope labeled prodrug d_3_-DHED) sophisticated in vivo pharmacokinetics and brain distribution experiments involving intravenous (i.v.) and oral (p.o.) administrations and liquid chromatography–tandem mass spectrometry (LC–MS/MS) measurements [23,36].

**Figure 6:**
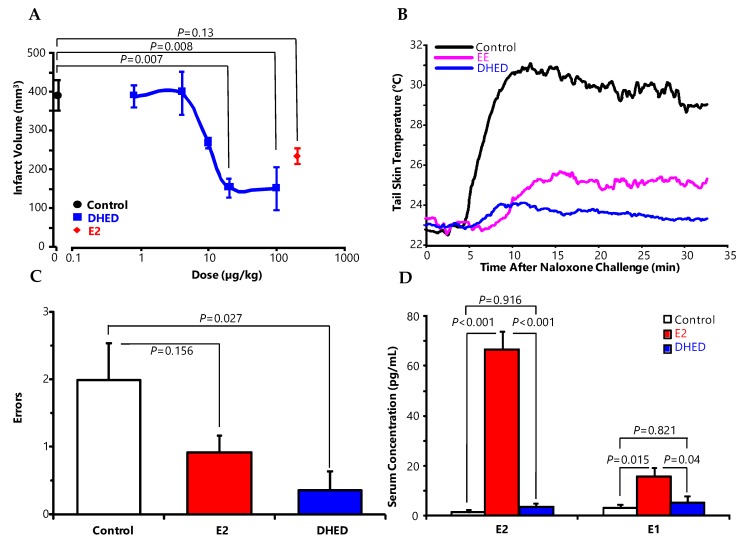
DHED treatment elicits profound neuropharmacological effects without increasing circulating estrogen levels. (**A**) Dose-dependent brain lesion volumes in ovariectomized (OVX) rats treated with DHED 1 h before tMCAO followed by 24-h reperfusion. The control groups received 200 µg/kg, s.c., E2 or vehicle alone. One-way ANOVA yielded a main effect of treatment (F_(6,14)_ = 8.76, *p* < 0.001). Data are average ± SEM, *n* = 3. Similarly to the rats treated with E2, statistical analyses revealed significant differences infarct volume of vehicle-treated animals, when rats received 20 and 100 µg/kg of DHED. (**B**) Tail-skin temperature changes in an OVX rat hot flush model after oral administrations of DHED, EE used as positive control, and the vehicle control. (**C**) In the delay match-to-sample plus maze test, E2- and DHED-treated middle-aged OVX Fisher-344 rats (continuously for 48 days by s.c. Alzet osmotic minipumps delivering 4 µg daily dose, respectively) made fewer errors compared to the vehicle-treated control group. One-way ANOVA: F(2,12) = 4.73, *p* = 0.031, *n* = 5 per treatment group. (**D**) Serum E2 and E1 concentrations of treatment groups from Panel **C**. Data are average ± SEM, *n* = 5. All displayed *P* values were determined by ANOVA followed by post hoc Student-Newman-Keuls multiple comparison tests, and *p* < 0.05 was considered statistically significant. Reproduced with permission from reference [36]; copyright American Association for the Advancement of Science.

The dose-dependent neuroprotective effect of E2 [36] when formed from DHED in the brain is shown in Figure 6A. Because of CNS-directed E2 delivery by DHED and the prodrug’s significantly improved physicochemical properties for brain uptake from the circulation relative to those of E2 (e.g., clogP of 1.67 versus 4.01 for the highly lipophilic parent E2 [36]), it is not surprising that approximately 10-fold higher E2 concentration was needed upon direct E2 administration to manifest the same degree of neuroprotection in an animal model of ischemic stroke (transient middle cerebral artery occlusion, tMCAO, followed by reperfusion. ED_50_ (dose equivalent to 50% of the maximum effect) for DHED treatment was estimated to be 15 µg/kg in this paradigm, while direct E2 treatment brought about ED_50_ of ~200 µg/kg.

Post-stroke treatment with DHED also resulted in profound neuroprotection with associated functional recovery [36], implicating that the injured brain is capable of retaining its ability to rapidly metabolize the prodrug to the neuroprotective parent estrogen. HEDD, the closely related bioprecursor prodrug derived from E1 (Figure 3B), has also been shown to provide significant neuroprotection in this stroke model without peripheral hormonal liability [35].

DHED also has excellent oral bioavailability, which is another important feature of our prodrug approach in the context of translational research [36]. Therefore, the clinically relevant oral route for DHED administration was successfully utilized to lower body temperature in a rodent model of menopause (Figure 6B) [23,36]. Because the parent E2 lacks appreciable oral bioavailability, the orally active synthetic estrogen, ethinyl estradiol (EE) was used as positive control in this paradigm. In another study shown in Figure 6C, continuous 7-week s.c. administration of DHED significantly decreased working memory errors committed by OVX middle-aged rats compared to control animals in a delay match-to-sample plus maze test without the increase in circulating estrogen levels (Figure 6D) and, therefore, without uterotrophic effect [36].

**Figure 7:**
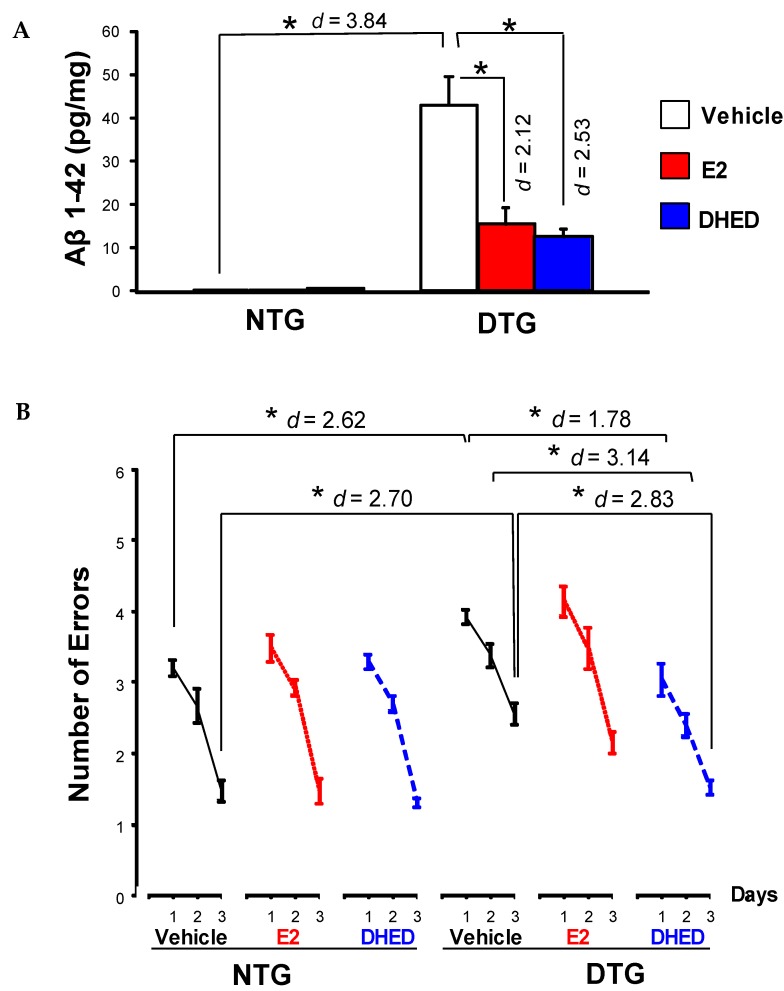
Evaluation of DHED treatment (8 weeks at 2 µg/day s.c., continuously by ALZET osmotic pumps) in males using the APPswe/PS1dE9 DTG (DTG) mouse model of AD. (**A**) Aβ 1–42 peptide levels in the brain of male non-transgenic (NTG) and DTG mice after treatment with vehicle, E2 (also 8 weeks at 2 µg/day s.c., continuously by ALZET osmotic pumps) and DHED; * *p* < 0.05 by ANOVA followed by post hoc Tukey’s honestly significant difference test, effect sizes indicated by Cohen’s d values. (**B**) Radial arm water maze (RAWM) results of cognitive testing. Number of errors are shown as averages for each day ± SEM (N = 7–9/group); within-group comparisons, indicating that each additional trial significantly improved response, are not displayed. DHED-treated DTG mice showed statistically significant improvement in ability to learn the task on days 1, 2 and 3 compared to vehicle-treated DTG controls, and performed like the NTG animals that manifested no behavioral impairment because they did not carry the APPswe/PS1dE9 transgene. Asterisks indicate *p* < 0.05 by repeated measures ANOVA followed by post hoc Tukey’s HSD test; effect sizes indicated by Cohen’s d values. Reproduced with permission from reference [51]; copyright Elsevier Inc.

We also have shown that DHED treatment decreased amyloid-β peptide (Aβ) levels in the brain of OVX and intact female APPswe/PS1dE9 double-transgenic (DTG) mice used as a model of Alzheimer’s disease (AD) [50]. Consequently, these animals had a higher cognitive performance, similar to those treated with the parent E2, when compared to the untreated control group. An independent follow-up study not only have confirmed these promising results, but also found that DHED treatment reduced oxidative and inflammatory stress, as well as decreased phosphorylated tau (τ) protein levels in OVX transgenic AD mouse [52]. An investigation focusing on assessing DHED’s therapeutic potential in males of the APPswe/PS1dE9 DTG-mouse AD model also revealed reduction of Aβ formation and protection against Aβ-associated cognitive impairment, as summarized in Figure 7 [51]. Therefore, DHED treatment could provide a remedy against early-stage AD, mimicked by the selected animal model of the disease in a gender-independent fashion. 

**Figure 8:**
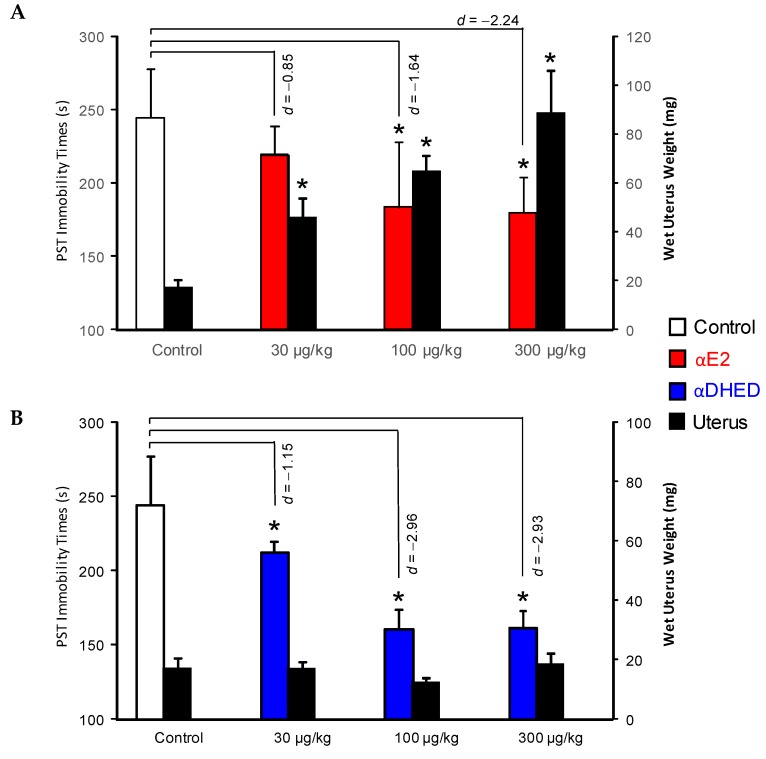
Immobility times and wet uterus weights after the s.c. administration (5 days, once daily) of increasing doses of (**A**) αE2 and (**B**) αDHED to OVX CD1 mice. Data are expressed as mean ± SD, *n* = 6, except for vehicle control group (*n* = 12). Asterisk (*) denotes statistically significant difference from control (one-way ANOVA followed by post hoc Dunnett’s tests); effect size of behavioral response is indicated by Cohen’s d. Adopted with permission from reference [37]; copyright American Chemical Society.

The isomeric biopecursor prodrug αDHED (Figure 3B), derived from αE2 (Figure 1), has also shown great selectivity towards the brain in terms of bioactivation to the parent drug [37]. We have utilized the well-known antidepressant effect of estrogens [11,46] to test αDHED’s ability to selectively deliver αE2 into the brain to exert this neuropharmacodynamic effect of the hormone. In the Porsolt swim test (PST), immobility time is associated with the “depressive state” [55], and PST is a useful paradigm for the initial screening for potential antidepressant agents. Here, both the direct administrations of αE2 (Figure 8A) and αDHED (Figure 8B) produced a dose-dependent reduction in immobility times, but αE2 treatment also triggered a profound uterotrophic effect (Figure 8A, black bars). However, αDHED treatment was devoid of this detrimental common side effect (Figure 8B, black bars) of current systemic estrogen therapies.

The measured αE2 contents [56] in relevant biological matrices, such as blood, brain and uterus (Table 1), also confirmed that systemic administration of αDHED produced αE2 only in the brain and not in the periphery [37]. As we have seen in the case of E2-CDs [46], here we have also confirmed that a high circulating level of an estrogen is associated with a high level of estrogen exposure to the uterus (Table 1) and a significant increase in wet uterine weight due to fluid imbibition (Figure 8A, black bars).

Soon after we had established the unique properties of estrogen-derived *para*-quinols in terms of brain-selective bioactivation to the corresponding estrogens (Figure 3) [35], we also became interested in their ophthalmic applications, especially in the context of ocular neuroprotection [57,58,59]. To survey the therapeutic safety of this additional DHED application, first we followed H_2_O_2_ production as a marker of DHED-derived potential neurotoxic side effect upon incubating DHED in rat retina homogenate [57]. The origin of excess H_2_O_2_ in a tissue is principally from superoxide radical-anion decomposition by superoxide dismutase [58]. Induction of oxidative stress by redox cycling of estrogen metabolites has been well-established [20]. In this redox cycling, *ortho*-quinones formed from catechol estrogens are critical players to trigger oxidative stress and, thus, cause neurotoxicity [60]. A profound prooxidant representative of these species is estra-1,5(10)-dien- 3,4,17-trione (3,4-E1 quinone), which was used as a positive control. As data in Table 2 show, conversion of DHED in rodent CNS tissues did not manifest toxicity using induction of oxidative stress as a marker.

As mentioned above, our particular interest in ophthalmic applications of DHED pertains to ocular neuroprotection. Currently, there are no effective pharmacotherapeutic interventions to prevent or halt this process. Irreversible retinal neurodegeneration is a fundamental pathological feature of several blinding eye diseases, such as glaucoma and age-related macular degeneration (AMD). Studies have shown similarity of ocular neurodegeneration with neurodegeneration of the brain, owing to several common mechanistic and biological contributors [61,62]. However, initiation and/or progression of neurodegeneration are yet to be fully understood, although aging is a definite risk factor [63]. Extension of our DHED prodrug approach for the neural retina and optic nerve was obvious, because estrogens have long been implicated in their health [13,14,64,65]. For example, the Rotterdam Study has found that early menopause (thus, a significant decline in endogenous E2) is associated with a higher risk of open-angle glaucoma [66]. Another report has speculated on the detrimental consequences of estrogen deficiency after menopause as a causative factor for glaucomatous damage with age [67]. In glaucoma, loss of retinal ganglion cells (RGCs) that transmit a vast amount of visual information from the retina to the brain has been identified as the earliest form of cell death, primarily by apoptosis [13,61]. This process eventually leads to loss of visual acuity.

In the routinely used Morrison model of glaucoma based on surgically-elevated intraocular pressure [68], we have previously shown that 3-week daily E2 eye drops resulted in significant E2 concentration in the retina (~22 ng/g) with concomitant profound neuroprotective therapeutic benefits [13]. The number of apoptotic cells in the RGC layer was significantly decreased when compared to vehicle-treated controls and, therefore, deterioration in visual acuity in these animals was markedly prevented. With LC-MS/MS-based proteomics, we have also provided an initial survey of the expression of various retinal proteins responding to the neuroprotective topical treatment that could be linked to important retinal functions with potential implications for neuroprotective ocular drug therapy. On the other hand, systemic E2 exposure could not be avoided with E2 eye drops, as confirmed by the measured high circulating estrogen levels (>400 pg/mL) and through assessing the uterotrophic effect brought about by chronic E2 eye drop treatments [13]. These findings highlighted the need for therapeutic safety and, thus, confirmed the utility of the topical application of our bioprecursor prodrug to deliver the powerful neuroprotectant E2 into the retina without peripheral hormonal liability [69].

Another blinding eye disease is (dry) age-related macular degeneration (AMD) that leads to pathological changes and dysfunction within the retinal macula and especially in the photoreceptor cells leading to central vision loss and blindness [70]. Akin to glaucoma, the pathological mechanisms underlying dry AMD remain unknown and are presumably a combination of genetic factors and oxidative stress, which critically contribute to the initiation and progression of the disease. Dry AMD-related processes of neurodegeneration are potentially triggered by interplay between two groups of factors: harmful external stimuli and intrinsic genetic and/or maladaptive responses by retina tissue and cells. The hallmark of dry AMD is the accumulation of fat and protein deposits termed drusen underneath the retina. A transgenic mouse model (MCP-1^−/−^) that exhibits, in part, some of the characteristics of AMD pathogenesis [71] was used to probe E2‘s ability to preserve photoreceptor cells upon topical eye drop treatments with our propriety bioprecursor prodrug of E2. Assessments of targeted drug delivery and in vivo neuroprotection in terms of preservation of visual functions were carried out analogously as reported before [13,69]. Routine histological assays were also used to assess neuronal viability and different stages of photoreceptors cell death. Our study has confirmed [69] that E2 effectively preserved visual function or reduced visual loss when compared to vehicle controls. Moreover, similarly to what we have seen in Figure 6A in an ischemic stroke model, prodrug treatment produced a more robust preservation of contrast sensitivity in the ipsilateral/treated eye (approximately 3-fold increase compared to the vehicle control) than the parent drug (approximately a 2-fold increase compared to the vehicle control). This effect was plausibly due to the significantly improved physicochemical properties of DHED compared to those of E2 [36] regarding drug diffusion through biological membranes including the cornea. This corresponded to approximately 75% and 50% preservation of contrast sensitivity compared to the wild-type (WT) control for prodrug and E2 treatments, respectively, while the contralateral/untreated eye lost approximately 80% of contrast sensitivity in relation to the WT control [69].

## 5. Conclusions

Ensuring selective delivery to the intended site of action and thereby avoiding unwanted off-target impact is the key to neurotherapy involving estrogens. This review focused on our novel bioprecursor prodrug approach for CNS-selective estrogen therapy. These prodrugs are derived from the conversion of the phenolic A-rings of the hormones to *para*-quinol structures, whose unique and distinguishing properties, together with site-specific expression of the target enzyme, allow for selective rearomatization to the corresponding estrogens exclusively in the CNS. Therefore, peripheral hormonal liability is avoided. We summarized the enabling chemistry and discussed applications in preclinical animal models to the potential treatment of several maladies associated with the deprivation of the CNS from estrogen, or respond to interventions by this hormone. With most of these applications addressing critically unmet medical needs, the hitherto unprecedented CNS-selectivity and promise of therapeutic safety warrant further development of the patented strategy from bench to clinic.

## Figures and Tables

**Table 1:** Estrogen concentrations in blood, cortex and uterus after s.c. treatment of OVX CD1 mice (*n* = 4) for 5 days, once daily with vehicle (control), αE2 (300 µg/kg body weight) and α-DHED (300 µg/kg body weight), respectively. Errors are given as SEM. Adopted with permission from reference [37]; copyright American Chemical Society.

Test Agent	Serum (ng/mL)		Cortex (ng/g)		Uterus (ng/g)	
	E2	αE2	E2	αE2	E2	αE2
Control	<0.01	N.D.	0.21 ± 0.07	N.D.	0.44 ± 0.06	N.D.
αE2	<0.01	5.4 ± 0.2	0.18 ± 0.02	1.8 ± 0.7	2.00 ± 0.45	9.0 ± 0.8
αDHED	<0.01	<0.01	0.28 ± 0.09	8.9 ± 1.7	0.37 ±0.10	N.D.

N.D.—not detected; E2 represents the endogenous E2 content in OVX mice.

**Table 2:** Rates of H_2_O_2_ production (nmol·h^−1^·mg protein^−1^, mean ± SD) at 37 °C in selected tissue homogenates (0.2%, *w*/*v*, pH 7.4) of OVX rats in the absence and presence of the prooxidant E2 metabolite, 3,4-E1Q and DHED (100 μM, respectively). * indicates statistically significant differences (ANOVA followed by post hoc Dunnett’s test, *p* < 0.05) from the respective controls. Adopted with permission from reference [57]; copyright Elsevier Inc.

Test Agent	Retina	Brain	Liver
Control	178 ± 3	155 ± 18	58 ± 16
3,4-E1-quinone	580 ± 50 *	1050 ± 8 *	687 ± 40 *
DHED	163 ± 9	125 ± 19	34 ± 18
